# CaSTLe – Classification of single cells by transfer learning: Harnessing the power of publicly available single cell RNA sequencing experiments to annotate new experiments

**DOI:** 10.1371/journal.pone.0205499

**Published:** 2018-10-10

**Authors:** Yuval Lieberman, Lior Rokach, Tal Shay

**Affiliations:** 1 Department of Life Sciences, Ben-Gurion University of the Negev, Beer-Sheva, Israel; 2 Department of Software and Information Systems Engineering, Ben-Gurion University of the Negev, Beer-Sheva, Israel; Universitatsmedizin Greifswald, GERMANY

## Abstract

Single-cell RNA sequencing (scRNA-seq) is an emerging technology for profiling the gene expression of thousands of cells at the single cell resolution. Currently, the labeling of cells in an scRNA-seq dataset is performed by manually characterizing clusters of cells or by fluorescence-activated cell sorting (FACS). Both methods have inherent drawbacks: The first depends on the clustering algorithm used and the knowledge and arbitrary decisions of the annotator, and the second involves an experimental step in addition to the sequencing and cannot be incorporated into the higher throughput scRNA-seq methods. We therefore suggest a different approach for cell labeling, namely, classifying cells from scRNA-seq datasets by using a model transferred from different (previously labeled) datasets. This approach can complement existing methods, and–in some cases–even replace them. Such a transfer-learning framework requires selecting informative features and training a classifier. The specific implementation for the framework that we propose, designated ''CaSTLe–classification of single cells by transfer learning,'' is based on a robust feature engineering workflow and an XGBoost classification model built on these features. Evaluation of CaSTLe against two benchmark feature-selection and classification methods showed that it outperformed the benchmark methods in most cases and yielded satisfactory classification accuracy in a consistent manner. CaSTLe has the additional advantage of being parallelizable and well suited to large datasets. We showed that it was possible to classify cell types using transfer learning, even when the databases contained a very small number of genes, and our study thus indicates the potential applicability of this approach for analysis of scRNA-seq datasets.

## Introduction

Single-cell RNA sequencing (scRNA-seq) is an emerging technology that measures, in a single experiment, the expression profile of up to 10^5^ cells, at the level of the single cell [1]. There are currently hundreds of scRNA-seq datasets in the public domain [2], and the number of new datasets is growing rapidly. Intensive attention has thus been devoted to addressing–by various methods [3]–the unique analytical challenges posed by the analysis of scRNA-seq datasets. The labeling of the cells (e.g., in terms of cell type, cell state, and cell cycle stage) in an scRNA-seq dataset that profiles a non-homogenous cell population is currently performed by one of two approaches, one experimental and the other computational, namely, fluorescence-activated cell sorting (FACS) or clustering the cells based on gene expression data, followed by manual annotation of each cell cluster. Both these approaches have inherent drawbacks. The first approach–FACS–requires an additional experimental step (beyond the actual sequencing experiment) and is limited in throughput, as it is necessary to track the cells, typically by sorting from the cell sorter to multiwell plates. This approach is thus not practical for new scRNA-seq methods, such as drop-seq [4], in which large numbers of cells are profiled. The second approach–clustering and manual annotation [5,6])**–**depends not only on a dimensionality reduction method [typically principal component analysis (PCA) or t-distributed stochastic neighbor embedding (t-SNE)] and a clustering algorithm used to define distinct cell types but also on the knowledge and arbitrary decisions of the annotator of each cell type. The labeling is therefore subjective. As a result, comparisons of cells of presumably the same cell type between experiments becomes complicated, if not impossible. In addition, the annotator typically uses knowledge of existing cell type markers. However, those known markers are defined and used at the protein level. RNA levels can explain about 40–80% of the variance in protein levels [7], meaning that reliable protein markers are not necessarily reliable markers at the RNA level. For example, natural killer cells express CD8a RNA, even though they do not carry CD8 protein on their cell surface. An additional drawback is that the inherently low sampling and noise in measurements at the single-cell level makes classification based on a small number of marker genes very inaccurate. Classification based on larger number of genes is much more robust to noise and sampling depth. Thus, although the labeling of cells of known cell types is, by definition, a supervised learning task, it is currently achieved by unsupervised methods with manual input. Recent attempts to address the above-described problems have led to the development of several different approaches for automatic annotation of cell types, including our own, which is presented in this article.

This work offers a new approach for labeling cells that comprises the direct re-use of a classification scheme that was learnt from previous similar experiments, namely, the machine learning concept known as “transfer learning” [8]. This classification approach can complement the labeling of cell types by FACS or clustering in a dataset that contains previously profiled cell types. It can also be applied in cases of cells that are in a transitional state between cell types, and it can aid in identifying contamination by other cell types. In scenarios where the source and target datasets are similar, the proposed method can replace clustering, thereby facilitating fast and objective identification of cell types, but with the drawback that it cannot detect novel cell types. To partially overcome this caveat, the method does identify cells that are not well classified into any of the predefined cell types, thereby highlighting those cells that are candidates for additional study. Although it is possible to apply standard classification algorithms on scRNA-seq datasets, a cell type classification algorithm for scRNA-seq datasets will not be useful in practice unless it can be applied to other (unlabeled) datasets that were not 'seen' during the model training. It might appear that this transfer of the model is simply a trivial addition of data instances to an existing dataset, but this is not so, since scRNA-seq datasets display different levels of noise and feature distribution due to wide variations in both experimental and technical aspects of scRNA-seq (cell barcoding, sequenced regions, library preparation, sequencing method, read depth, etc.). For example, if we consider two datasets in which 10^6^ reads are sequenced per cell in one dataset and 2*10^6^, in the other, the expression level of each gene in the second dataset will be doubled; this difference in expression levels can easily be accounted for, but more problematically, there will be in an increase in the number of lowly expressed genes whose RNA has been sequenced. Thus, the technical variability between datasets raises the need for employing a more sophisticated method for transfer learning. To the best of our knowledge and based on repositories of scRNA-seq methods (awesome-single-cell [9] and scrna-tools.org [10]), there are currently no methods that apply the specific concept of transfer learning to use information from one scRNA-seq dataset to annotate another, even though machine learning methods have indeed been applied in scRNA-seq analysis, e.g. [6,11]. For example, the classification methods listed in scrna-tools.org [10] derive from a variety of different concepts, but they are all limited to specific cases. DistMap [12] is based on spatially distributed scores, making it relevant only for in-situ scRNA-seq. MetaNeighbor [13] assesses the replicability of cell types across datasets but does not annotate cells. Finally, in singleR [14], CellSearchAtlas [15], and scmap [16], annotation is based on similarity to a predefined reference set of cell types.

To exploit and to illustrate the power of transfer learning, we have developed a method that we designate ''CaSTLe–classification of single cells by transfer learning'' for assigning a cell type to each cell in an experiment. This method is based on an elaborate workflow of univariate feature engineering steps, as described below, followed by application of a pre-tuned XGBoost [17] classifier. The feature engineering steps include: selecting genes with the top mean expression and mutual information gain, removing correlated genes, and binning the data according to pre-defined ranges. All these steps ensure that the source and target datasets are brought to a common denominator that will allow accurate transfer of the classification model.

## Results

### Design objectives

The steps of CaSTLe were formulated following a comprehensive survey of supervised methods for classifying scRNA-seq datasets with the aim of elaborating a workflow that is accurate, efficient, and consistent. The first objective–classification accuracy–was evaluated against previously labeled datasets, where the goal was to correctly classify as many instances as possible. When performing a multi-class classification, i.e., classifying all cell types in a single step, we assumed that the source dataset contained all the cell types that are profiled in the target dataset. In this scenario, accuracy was measured as the percentage of instances correctly classified. When we assume that the target dataset may contain cell types not profiled in the source dataset, we can divide the classification into several steps, classifying each cell type in a separate step by using binary-class classification. This allows us to use the measures of Sensitivity, Specificity and AUC, where: Sensitivity is measured as the percentage of cells in the target dataset that were identified as being of a particular cell type; Specificity is measured as the percentage of true positives out of the cells that were classified as the cell type; and AUC is defined as the area under the receiver operating curve, namely, the tradeoff between Specificity and Sensitivity. The second objective–efficiency–namely, the ability to run the entire process using a personal computer in a reasonable time of a few minutes, was required for establishing the utility of the approach of transfer learning for analyzing large datasets of tens of thousands of cells. The third objective–consistency–was required for achieving high accuracy for a wide range of datasets without the need to adjust the parameters of the algorithm according to the specific properties of each dataset. It is possible that this constraint can be relaxed in the future, if a means can be found to optimize the parameters of the algorithm according to dataset properties.

### Method details

As mentioned above, CaSTLe, the proposed method for classifying scRNA-seq datasets by using transfer learning, is based on a series of simple univariate feature selection methods, followed by application of a pre-tuned XGBoost classifier. The workflow comprises: selecting genes with the top mean expression and mutual information gain, removing correlated genes, binning the data and running XGBoost to build the classifier, as detailed below in the following steps:

1. Retrieve both datasets–source and target–and prepare a dataframe, in which columns are features (genes) and rows are instances (cells).
2. Remove the features that appear only in one dataset.
3. Remove rarely expressed genes, namely, features with non-zero values in less than 10 cells.
4. Select the 100 features with the highest overall mean. For each gene, sum the expression values in the two datasets and divide the sum by the number of cells in both datasets.
5. In the source dataset, select the 100 features with the highest mutual information between the features and the class. Since mutual information is calculated on two nominal variables, we split the features into bins of [0], (0,1], (1, 6], (6, ∞).
6. Unite the 200 features from steps 1 and 2, resulting in a set of 100–200 unique features, depending on the overlap between the two groups.
7. Remove the features with high inter-feature correlation, namely, Pearson correlation higher than 0.9. For each pair of correlated genes, remove one arbitrarily.
8. Convert the features from continuous values to four ordinal bins using the range: [0], (0,1], (1, 6], (6, ∞).
9. Remove features for which all values fall in the same bin. This step removes a very small number of features, if any at all.
10. Build an XGBoost classification model on a random 80% of the source dataset. Use pre-tuned parameters: eta = 0.7, nround = 20, gamma = 0.001, max_depth = 5, min_child_weight = 10.
11. Evaluate the model on the held-out 20%. If classification accuracy is not satisfactory, consider improving the model by tuning the parameters.
12. Classify the target dataset using the model learnt on the 80% of the source.

It should be noted that all steps in this process can be parallelized. It is trivial to parallelize steps 2–8, since each step consists of many tasks that are not dependent on the results of other tasks. Steps 9 and 10 can be parallelized as part of the XGBoost algorithm implementation. It should also be noted that the justification for using both the highest mean and the highest mutual information (steps 4 and 5) was empirical, as it helped to improve the accuracy in some cases.

The full source code in R is available in GitHub, a public code repository, at https://github.com/yuvallb/CaSTLe.

### Tested datasets

CaSTLe was developed on three scRNA-seq datasets and tested using nine scRNA-seq datasets. All datasets used are results of real experiments (not synthetic data) that are publicly available at NCBI GEO [18] or ArrayExpress [19]; see the “Materials and Methods: Datasets” section below for full details. All datasets contain gene expression data, where each cell is labeled with a cell type. The cell type label from each dataset was used as ground truth for evaluating the accuracy of the CaSTLe method. From these nine datasets, a total of 12 source-target combinations was evaluated. These 12 combinations featured a wide range of properties, such as: number of cells, number of cell types, cell type distribution ratio and sparsity of data (Table 1) and thus represent the heterogeneity of the public scRNA-seq datasets.

**Table 1:** Test datasets.

Dataset number	Group^1^	Accession	Classes	Number of cells	Number of genes	Imbalance ratio^2^ (majority/ minority)	Sparsity^3^ (% zero values)
**1**	HSCs	GSE59114 [20]	3	1,428	8,422	1.5	57.4
**2**	HSCs	GSE59114 [20]	3	564	19,586	1.8	62.8
**3**	HSCs	GSE81682 [21]	3	1,920	34,892	3.9	24.9
**4**	Retina	GSE63473 [4]	3	37,309	23,288	18.1	95.3
**5**	Retina	GSE81904 [22]	3	26,530	13,166	258.2	93.1
**6**	Embryo	GSE45719 [23]	5	256	22,431	9.5	40.8
**7**	Embryo	E-MTAB-3321 [24]	5	124	41,427	10.7	28.4
**8**	Pancreas	GSE81608 [25]	2	1,358	39,851	1.9	71.7
**9**	Pancreas	E-MTAB-5061 [26]	2	1,156	25,525	3.3	64.5

^1 ^See Materials and Methods for an explanation of HSCs, Retina, Embryo and Pancreas.

^2^ Calculated as the number of samples in the majority class divided by the number of samples in the minority class.

^3^ Calculated as the percentage of zero values, before any preprocessing and feature selection.

The nine datasets used in the work were grouped according to the cell types that they profile. Each group contained two or three comparable datasets, containing similar cell types (Table 1).

Out of these nine datasets, six pairs contained the same cell types, so they could be used to evaluate transfer learning (Table 2).

**Table 2:** Dataset pairs used for transfer learning.

Dataset number	Dataset pair^1^	Organism	Classes	Class type	Number of common genes	Distribution similarity (cosine similarly of class distribution)
**1**	HSCs 1 to 2	Mouse	3	Ordinal	7,423	0.97
**2**	HSCs 1 to 3	Mouse	3	Ordinal	7,423	0.85
**3**	HSCs 2 to 3	Mouse	3	Ordinal	7,423	0.77
**4**	Retina	Mouse	3	Nominal	12,307	0.22
**5**	Embryo	Mouse	5	Ordinal	12,783	0.32
**6**	Pancreas	Human	2	Nominal	16,299	0.98

^1^See Materials and Methods for an explanation of HSCs, Retina, Embryo and Pancreas.

Each of the four groups of datasets represents a unique characteristic:

- The hematopoietic stem cells (“HSCs”) group contains experiments from different mouse strains, and shows, in practice, very high levels of technical (or other) differences. These differences are also reflected in the wide range of the numbers of features (8,422–34,892). Since the number of actual genes is similar, the gap between the highest (34,892) and lowest (8,422) numbers of features reflects different preprocessing steps performed on the three datasets—probably the use of different libraries. The classes in this group are ordinal, representing consecutive stages of immune cell differentiation.
- The “Retina” group contains the highest number of samples, the highest rate of zero values and the highest imbalance ratios. This group also shows the highest difference in distribution between datasets, measured using the cosine similarity between the vectors of number of instances in each class of each dataset (Table 2). Dataset 4 contains 6,285 bipolar cells, 1,624 Müller cells and 29,400 rod cells, while dataset 5 contains 23,494 bipolar cells, 2,945 Müller cells and only 91 rod cells. This means there is a large difference in the class distribution between the source and target, so it may be more difficult to transfer a model from one dataset to the other. To quantify the difference, we can compare the class distribution using the cosine similarity between (6285, 1624, 29400) and (23494, 2945, 91), which is as little as 0.22. The other dataset pairs range from 0.32 to 0.98, indicating that the class distribution difference is the highest in the Retina group.
- The “Embryo” group contains the smallest number of samples and the highest number of classes. The classes in this group are ordinal, representing early stages of embryonic development (2 cells, 4 cells, 8 cells, 16 cells and blastocyst). It should be noted that 'stages’ are not the same type of label as 'cell types,' and at a given stage a given cell may be more similar to a cell in the previous stage or in the next stage than to any cell from the same stage. However, we hypothesize that classification by stage may extract a differentiation signature. Nonetheless, this group is by far the most difficult to classify.
- The “Pancreas” group represents a two-class classification problem. This group is the only group to contain human cells, which can introduce many confounding factors and noise into the data, as humans are genetically different from one another, as opposed to mice from the same strain, which carry the same genome sequence.

### Transfer learning results–comparison to a ground truth measure

**Fig 1:**
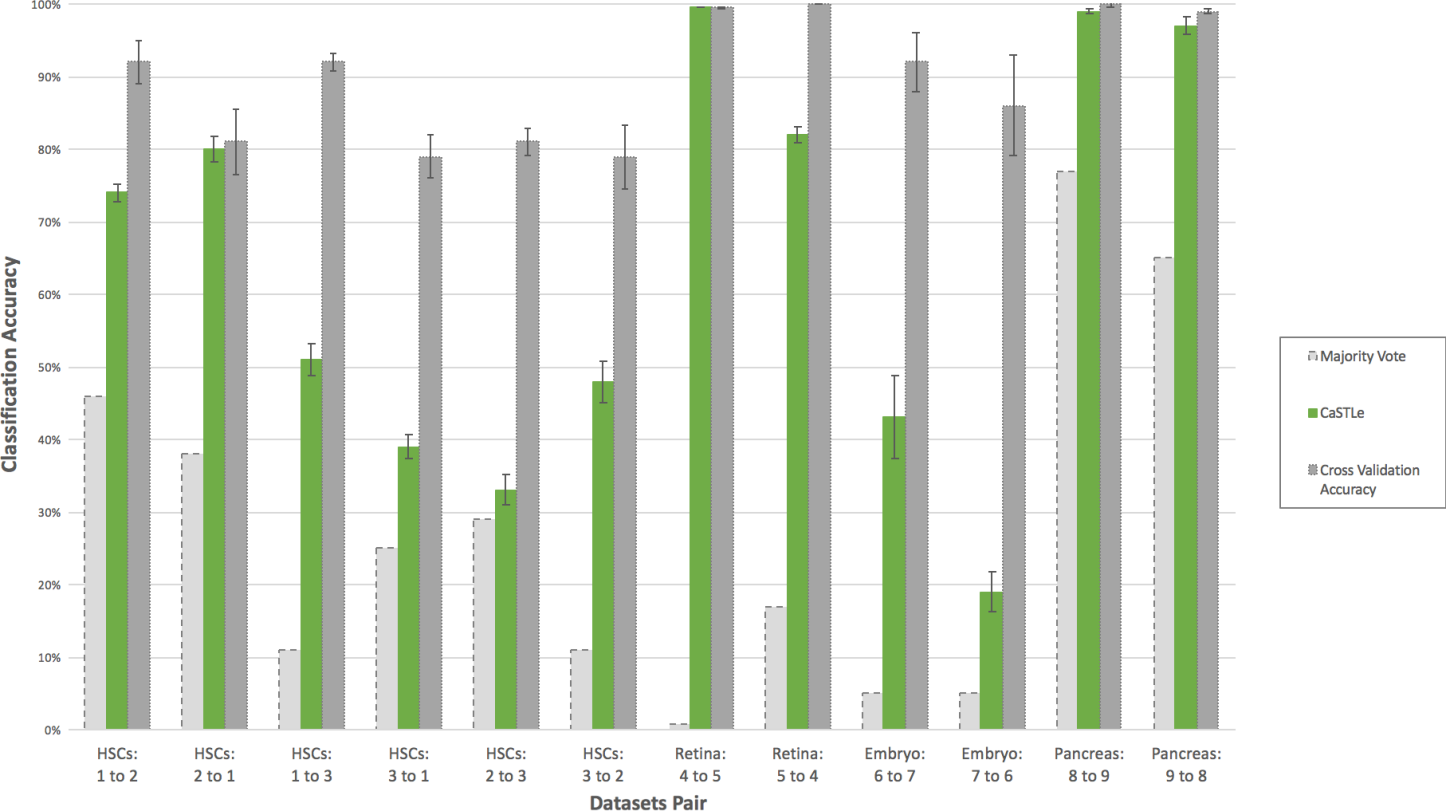
Classification accuracy of transfer learning by CaSTLe compared to the cross-validation accuracy (upper bound) and majority vote. Results are shown for the 12 source-target datasets pairs tested (X-axis). Error bars reflect the standard deviation between ten runs (irrelevant for majority vote).

The first evaluation of CaSTLe was performed against the majority vote classifier (classifying all target instances as the majority class of the source) and the cross-validation accuracy (validation on the same source dataset). In this step, we assumed that all cell types in the target dataset are contained in the source dataset. It is expected that a transferred model will be more accurate than a majority vote but not as accurate on the target dataset as its cross-validation accuracy on a held-out set. In our case, each model was trained on 80% of the source dataset and evaluated twice, first on the remaining 20% and then on the entire target dataset. This process was repeated ten times, and the accuracy measures presented in Fig 1 show the averages and standard deviations for these ten runs. Of note, using all the training set to train the classifier did not improve the results significantly, and thus we chose, for sake of simplicity, to use only the 80%, making the classifier accuracy on the source and target datasets comparable. The ground truth measure, which is used to calculate classification accuracy, is the labeling supplied with the published target dataset, which is typically based on manual annotation of the clusters from clustering after dimensionality reduction with PCA or tSNE (see more details in the “datasets” sub-section of the “Materials and Methods” section below).

As expected, the accuracy of CaSTLe in all 12 cases was lower than or the same as the upper bound of the cross-validation accuracy (Fig 1). The lower bound of the transfer classification, the majority vote classifier, was calculated as the percentage of instances in the target dataset that belong to the majority class in the source dataset. Reassuringly, the proposed method always outperformed the majority vote. Two particularly striking cases were HSCs 2 to 3 and Embryo 7 to 6 where the gap was very small, being 4% and 14%, respectively, which may indicate some room for improvement.

### Comparison of CaSTLe to other transfer learning methods

To evaluate the performance of CaSTLe against other benchmark methods, we compared it to two simple, yet commonly used, methods, each composed of a feature-selection phase and a classification phase. In this stage, too, we assumed that the source and target datasets contained the same cell types. The first benchmark method comprises selecting the top five features with the highest mean across both datasets and building a linear regression classifier on these features. The second benchmark method, representing an approach that is more specific to scRNA-seq datasets, comprises selecting the top 10 features according to the beta-Poisson single cell differential expression algorithm [27] and building a linear regression classifier on these features. The use of a linear regression for benchmark classification was chosen to represent a very simple and robust approach. For binary classification (Pancreas datasets), the regression was performed on 0/1 targets. For multi-class classifications, a one-against-all approach was chosen, selecting the class with the highest prediction as the predicted class. Here, too, each model was trained on 80% of the source dataset and evaluated twice, first on the remaining 20% held-out from the source dataset and then again on the entire target dataset. This process was repeated 10 times, and the reported accuracy is an average of these 10 models.

**Fig 2:**
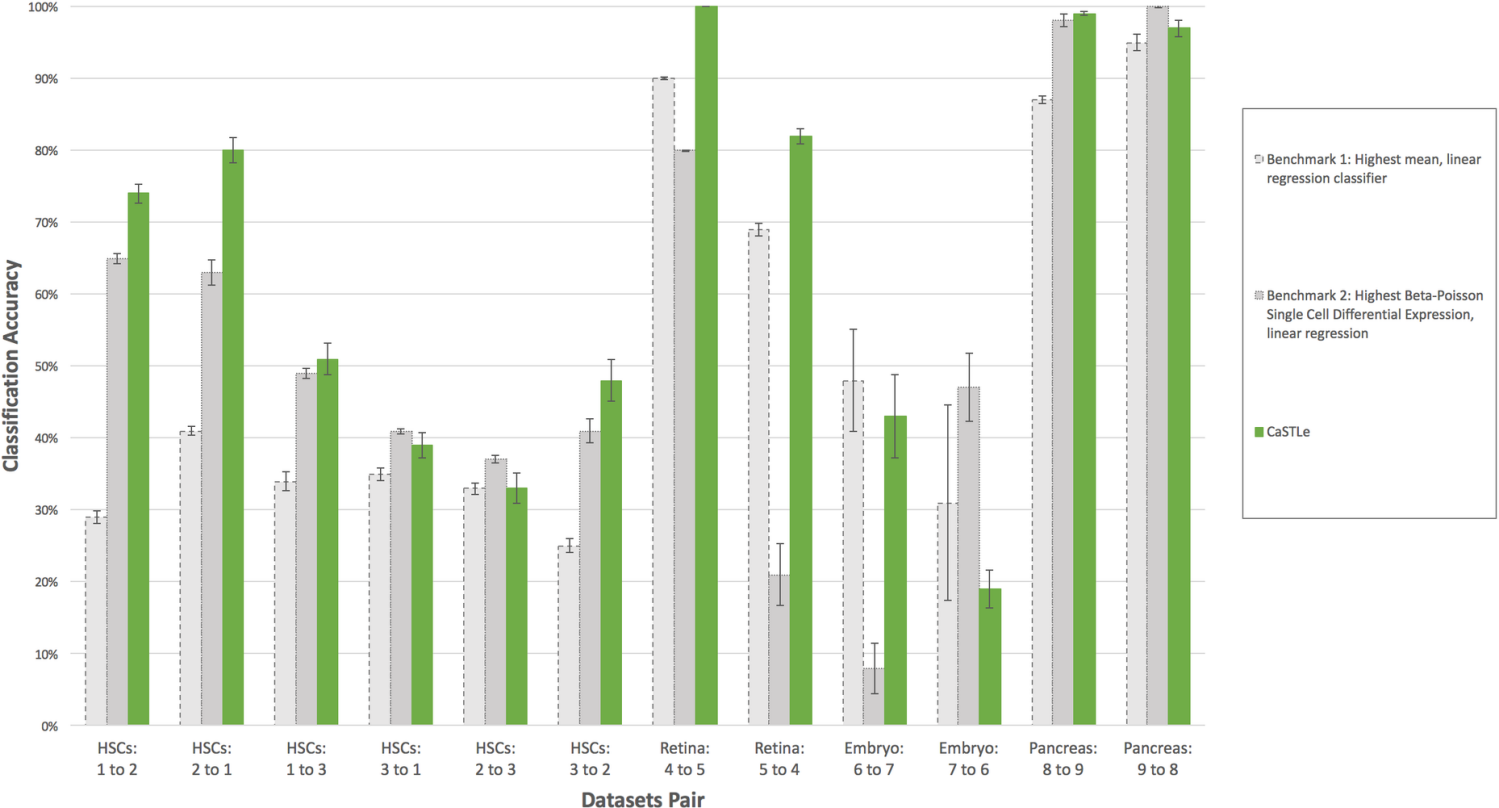
Classification accuracy of transfer learning by CaSTLe compared to two benchmark methods. Results are shown for the 12 source-target datasets pairs tested (X-axis). Error bars reflect the standard deviation between ten runs.

CaSTLe outperformed the first benchmark in all but two cases (Fig 2). In the case of Embryo 6 to 7, the benchmark classifier outperformed CaSTLe by 5% (p = 0.02; 1-tailed paired t-test), and in the case of HSCs 2 to 3, the average classification accuracy was the same, but the p value was very high (p = 0.27), indicating the results to be inconclusive. In all other cases, CaSTLe significantly outperformed the first benchmark (p<0.01; 1-tailed paired t-test).

The second benchmark method, which derives from a metric dedicated to single cell datasets, performed better than the first benchmark for the HSCs and Pancreas datasets, but worse for the Retina and Embryo datasets (Fig 2). This finding can, in itself, be seen as a weakness of the benchmark classifiers, since an ideal method should perform well in all cases (or alternatively stipulate the conditions for applying a model according to some dataset properties).

The evaluation of CaSTLe against the second benchmark showed that it outperformed the second benchmark in only 8 out of 12 cases: Benchmark 2 outperformed CaSTLe when learning from dataset HSCs 3 to dataset HSCs 1 by 2%, from HSCs 2 to HSCs 3 by 4%, from Pancreas 9 to 8 by 3%, and from Embryo 7 to Embryo 6 by 28%, the largest gap in learning. In all comparisons, the difference between accuracies was significant (p<0.01).

### Evaluation of CaSTLe performance per cell type

**Fig 3:**
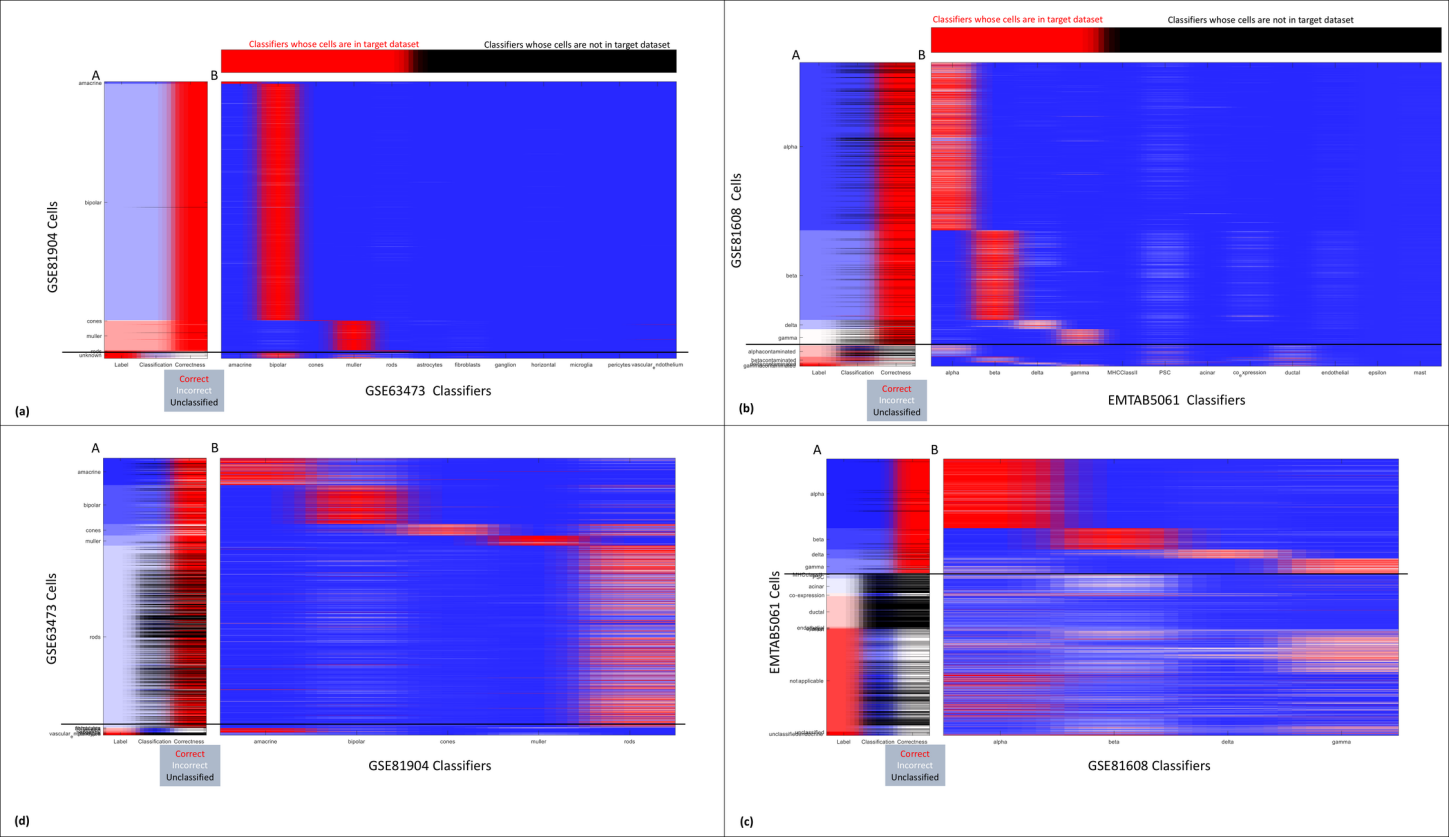
Classification of a target dataset by multiple binary classifiers. (A) The label of each cell in target dataset (left), the label each cell was given by the classifiers, black for unclassified (center), and the correctness of classification (right). (B) Heatmap of the scores given to each cell by each classifier, from zero (blue) to one (red). Horizontal black line separates the labels of those classifiers from labels for which classifiers were not trained. (a) Classification of target dataset GSE81904 by multiple binary classifiers built on source dataset GSE63473. Top bar shows which classifiers classified for labels that are in the target dataset. Note that many unknown cells were classified as bipolar or muller, which may be correct. (b) Classification of target dataset GSE81608 by multiple binary classifiers built on source dataset EMTAB5061. Top bar shows which classifiers classified for labels that are in the target dataset. Note that the labels that some of the seemingly incorrect results are very likely correct—the cells labeled as 'alpha contaminated' are classified as alpha or ductal, and same for beta, gamma and delta contaminated. (c) Classification of target dataset EMTAB5061 by multiple binary classifiers built on source dataset GSE81608. Note that many of the 'novel' cell types (acinar, ductal) were not classified, thus 'identified as novel'. Many of the incorrect cells are labeled 'coexpression' or 'not applicable' or 'unclassified', meaning that their classification may be correct. (d) Classification of target dataset GSE63473 by multiple binary classifiers built on source dataset GSE81904.

In the scenario presented above, we assumed both source and target datasets contain the same cell types. This assumption was made in order to prove that the method of transfer learning is viable. Using a multi-class classifier, we could evaluate the process performance using accuracy as a simple single measure. In the current stage, however, we no longer assumed that the source and target datasets contained the exact same cell types, as this assumption does not always hold true in the real world. The possibility of additional cell types in the target dataset will require a minor adjustment in the classification process. Regarding steps 1–12 specified in the “Method details” section above, for evaluating CaSTLe performance per cell type, we performed steps 1–4 once, and repeated steps 5–12 multiple times, once for each cell type present in the source dataset. See Fig 3A-3D for a graphic representation of the results in a heatmap. Since we were thus performing a single binary class classification, we could use more elaborate performance measures, such as Sensitivity, Specificity and AUC. We evaluated the method using dataset pairs 4,5 and 8,9, since these pairs contained both different cell types and the common cell types. Table 3 details the classification results for using CaSTLe as a series of binary classifiers, one for each cell type in the source dataset. For each classifier, we provide the percentage of instances in the source and in the target datasets (to indicate class imbalance), and the accuracy, sensitivity, specificity and AUC of the classifier. Each target dataset was classified for each cell type in the source dataset. Cell types that were annotated as unknown, not applicable, unclassified or contaminated were not used in the source dataset. We did however leave these cells when the dataset was evaluated as a target dataset. This can lead to a possible application of CaSTLe: helping to verify and identify types of cells that were not identified by other methods. For example, dataset GSE81904 contains 669 cells that are originally annotated as “unknown”. Using CaSTLe we were able to reach a classification with a probability higher than 90% for 550 cells out of these 669 (Fig 3A). An additional example can show how CaSTLe can be used to identify contaminated cells: dataset GSE81608 contains 31 cells annotated as “beta contaminated”. Out of which, 7 cells were classified as “beta” with a probability higher than 90%, and 20 cells had less than 50% probability of being classified as “beta” (Fig 3B).

**Table 3:** CaSTLe performance per cell type.

Cell type	Instances in Source	Instances in Target	Accuracy	Sensitivity	Specificity	AUC
**Mouse Retina: Learning from GSE63473 to GSE81904**						
Amacrine cells	9.9%	0.9%	99.9%	99.9%	94%	99.7%
Astrocytes	0.1%	None	99.9%	99.9%	-	-
Bipolar cells	14%	85.4%	97.5%	84.8%	99.7%	95%
Cones	4.2%	0.2%	99.9%	100%	93.7%	100%
Fibroblasts	0.2%	None	99.8%	99.8%	-	-
Ganglion cells	0.9%	None	99.9%	99.9%	-	-
Horizontal cells	0.6%	None	99.9%	99.9%	-	-
Microglia	0.1%	None	99.9%	99.9%	-	-
Müller cells	3.6%	10.7%	99.2%	99.1%	99.6%	99.9%
Pericytes	0.1%	None	99.9%	99.9%	-	-
Rods	65.6%	0.3%	99.9%	99.9%	96.7%	99.2%
Vascular_endothelial cells	0.6%	None	99.9%	99.9%	-	-
**Mouse Retina: Learning from GSE81904 to GSE63473**						
Amacrine cells	0.9%	9.9%	95.9%	97.9%	77.7%	96.2%
Bipolar cells	87.6%	14%	94.1%	95.2%	87.1%	96.4%
Cones	0.2%	4.2%	98.7%	99.8%	71.4%	99.5%
Müller cells	10.9%	3.6%	99.4%	99.5%	96.4%	99.6%
Rods	0.3%	65.6%	65.2%	90.3%	52.1%	88.6%
**Human Pancreas: Learning from GSE81608 to EMTAB5061**						
Alpha cells	59.4%	25.2%	88.8%	85.8%	97.7%	96.9%
Beta cells	31.6%	7.7%	94.1%	93.8%	97.8%	98.8%
Delta cells	3.3%	3.2%	95.7%	96.3%	85.1%	98.5%
Gamma cells	5.7%	5.6%	85.4%	85.6%	82.2%	94.9%
**Human Pancreas: Learning from EMTAB5061 to GSE81608**						
MHC class II cells	0.2%	None	100%	100%	-	-
Pancreatic stellate cells	2.5%	None	99.1%	99.1%	-	-
Acinar cells	8.5%	None	99.8%	99.8%	-	-
Alpha cells	40.9%	55.4%	90.6%	97.5%	85.1%	98.7%
Beta cells	12.5%	29.5%	97.3%	98.8%	93.9%	99.5%
Co-expression	1.8%	None	99.6%	99.6%	-	-
Delta cells	5.3%	3.1%	98.6%	99.6%	65.3%	99.1%
Ductal cells	17.8%	None	98.7%	98.7%	-	-
Endothelial cells	0.7%	None	100%	100%	-	-
Epsilon cells	0.3%	None	100%	100%	-	-
Gamma cells	9.1%	5.3%	97.7%	99.5%	64.7%	99.7%
Mast cells	0.3%	None	100%	100%	-	-

Though CaSTLe as a supervised method cannot identify novel cell types, it does enable identification of potential cells of previously unknown cell types, defined for this purpose as cell types not in the source dataset. Following the application of a series of binary classifiers, each cell is assigned to the cell type for which it got the largest score, assuming this score is above a predefined threshold. Cells whose maximal score is below this threshold are potentially of unknown cell types. To estimate the accuracy of unknown cell type identification, we tested the assignment of cell type for cells in the target dataset that belong to cell types that are not in the source dataset. For example, when learning from GSE81608 to EMTAB5061 (Fig 3C), when setting the threashold on 80%, out of 2047 such cells, 1732 (85%) were not classified, thus annotated as 'unknown cell type'. Only 265 (18%) of cells of a known cell type were given this annotation.

### Running times

To illustrate that CaSTLe is efficient and fast, we provide here some actual running times. All the run times below were measured without doing any parallelization, so they basically act as an upper bound that can be easily improved. The run times were measured on a MacBookPro (i7 16GB RAM), assuming that the data was already loaded into the memory (time to read a dataset file from the disk was not measured). Table 4 shows run times for each dataset pair twice: once for a multiclass classification, and the other for a per cell type binary classification, the time for later being higher, since it required repeating steps 5–12 for each cell type in the source dataset.

**Table 4:** Method runtimes.

Source dataset	Target dataset	Multi class classification–time (seconds)	Per cell type binary classification–time (seconds)
1	2	9	21
2	1	6	13
1	3	12	26
3	1	12	27
2	3	7	15
3	2	10	23
4	5	847	3055
5	4	794	1162
6	7	8	26
7	6	5	20
8	9	57	72
9	8	73	194

## Discussion

We showed that it is possible to classify single-cell RNA sequencing gene expression data in terms of cell types according to an independent labeled dataset containing similar cell types. CaSTLe, the method we developed for this process, is composed of a robust selection and transformation feature, followed by XGBoost classification. This method was shown to be parallelizable, efficient, and consistent across various test cases. For the multi-class scenario, in 10 out of 12 cases, CaSTLe outperformed a simple benchmark of highest mean features and linear model classification. In 8 out of 12 cases, CaSTLe outperformed a more sophisticated benchmark, the beta-Poisson single cell differentially expressed genes and linear model classifier. The strength and robustness of CaSTLe, compared to the two benchmark methods, was demonstrated by the high accuracy levels achieved for the larger and more imbalanced datasets. For the binary-class scenario, out of 18 cell types that appeared both in the source and target datasets, AUC values above 95% were obtained for 16 cell types. Out of 15 cell types that appeared only in the source dataset, a sensitivity higher than 97% was obtained for all 15 cell types, which means that an erroneous cell-type identification was probably made for as little as < 3% of cells. The performance in this stage was much better than in the multi-class classification, probably since we performed steps 5–9 separately for each cell type.

In general, it seems that the weakness of the proposed method was most striking for the two Embryo datasets. These two datasets were by far the smallest examined here (256 and 124 instances) and contained the largest number of classes (Table 1). In addition, as discussed above, the classification into embryonic development days is not necessarily inherent in the data. These findings can indicate that CaSTLe is suitable for large datasets and for classifying cell types. The Retina datasets, which, at the other extreme, were the largest examined (37,309 and 26,530 instances), exemplify the strength of the proposed method, achieving the highest accuracy by a large difference. Another important property of the Retina datasets was the highest imbalance (Table 1) and largest difference between class distributions (Table 2). The proposed method was able to overcome these difficulties and outperform the benchmarks.

A major advantage of CaSTLe is its high performance and scalability. All feature engineering steps are trivial to parallelize, since each step consists of many tasks that are not dependent on the results of other tasks. The learning of the classification model can be parallelized as an integral part of the XGBoost algorithm implementation. All the above make CaSTLe especially suitable for very large datasets.

The promising results obtained in this study indicate that future work is warranted to further refine and extend the method: other aspects of the data, not just cell types but also factors such as gender and age, should be classified, and a sufficient number of datasets should be evaluated to determine the conditions under which such a transfer classification is possible. It is our belief that the conditions for successful transfer depend on the differences in the protocols used for sequencing, the distribution of the target classes, the sparsity of the data and the number of instances (cells). Future research should thus be performed to explore and validate these conditions. Such research may also contribute to improving the transfer classification accuracy by adjusting the model to the dataset properties.

Several other directions can be explored to improve the classifier accuracy: the first involves weighting instances to give higher weight to source instances that are more similar to the target; that is, if the source dataset includes heterogeneous instances that are labeled by label L, and only some of those instances are similar, based on some similarity measure, to instances in the target dataset, then the classifier can be refined to classify only those instances as label L, omitting the other L instances from the training. Another possible improvement that can be explored is performing some of the preprocessing stages (read alignment, expression quantification) in house when possible, which could contribute to bridging the differences between datasets that result from using different preprocessing pipelines and transcriptome annotation versions. Another promising research direction is transfer classification where the target dataset is partially labeled. We believe that such a setting would improve the classification accuracy, but the exact technique to be applied remains to be explored. Ultimately, we envision a repository of classifiers for each cell type and state in each organism, which can be applied for annotating known cell types and highlighting new cell types and states.

## Materials and methods

### Datasets

Datasets were chosen on the basis of data availability, because we needed pairs of datasets that include a similar set of cell types, so that it makes sense to apply transfer learning to the pair. This research was performed and evaluated on nine datasets, organized into four groups of comparable datasets, enabling six pairs of datasets, as follows:

The first group profiled hematopoietic stem cells (HSCs) in two mouse strains from two datasets from GEO accession GSE59114 [20] and a third from GEO accession GSE81682 [21]. All three datasets contain scRNA-seq data for mouse HSCs in three differentiation stages: long-term HSCs (LTHSC), short-term HSCs (STHSC) and multipotent progenitor cells (MPP), which were regarded as the classification target classes. The datasets are also divided into populations of young and old mice and differentiate between the specific mouse strains sampled, factors that were not taken into consideration in the current study. Cell types were annotated using FACS [21] or clustering methods using PCA analysis [20].

The second group of datasets encompasses mouse retina cells, out of which three cell types, namely, bipolar, Müller glia and photoreceptor rod cells, were selected for evaluation. Other cell types were omitted from this validation, because they did not appear in both datasets or because they appeared in very low numbers. In the “Evaluation of CaSTLe performance per cell type” section we included all cell types with the aim to evaluate a more realistic use-case. This group consisted of the two largest datasets examined: GSE63473 [4] and GSE81904 [22]. In addition, this group showed the highest levels of imbalance and the greatest differences in class distribution between the two datasets (the minority class of one dataset is the majority class of the other). Cell types were annotated using tSNE followed by manual identification of cell groups based on the expression level of genes coding for commonly used cell surface markers [4] [22].

The third group of datasets, GSE45719 [23] and E-MTAB-3321 [24], consists of mouse embryonic cells in very early developmental stages. These datasets contain the smallest number of cells from the test datasets used in this research (Table 1) and are classified into five classes according to the developmental stage of the embryo: 2 cells, 4 cells, 8 cells, 16 cells and blastocyte. These datasets were retrieved from the Hemberg Lab Collection website, as detailed below.

The fourth pair of datasets, GSE81608 [25] and E-MTAB-5061 [26], profiles human pancreatic cells. From these datasets, a subsample containing only alpha and beta cells was extracted with the aim to allow us to represent a binary classification problem. In the “Evaluation of CaSTLe performance per cell type” section we included all cell types, again with the aim to evaluate a more realistic use-case. These datasets were also retrieved from the Hemberg Lab Collection website. Cell types were annotated using tSNE followed by manual identification of cell groups based on the expression level of genes coding for commonly used cell surface markers [25] [26].

### Dataset preprocessing

The data for datasets 1–3 (HSCs) was downloaded from the NCBI GEO website [18] and converted from CSV to an R dataframe. For all the datasets, we verified that the gene expression data was on a logarithmic scale. If not, we converted it to a log2 scale by applying the function *x = log2(x+1)*. The justification for this conversion being the common practice that reflects the log-normal distribution of the expression [28].

The data for datasets 4–9 was downloaded from a collection of publicly available datasets used by the Hemberg Group at the Sanger Institute [29]. The datasets were retrieved from that website in “Scater” object format [30], which was loaded for processing in R. The code used for all preprocessing stages performed on these datasets was publicly available in the github repository referenced in the Hemberg Lab Collection website: https://github.com/hemberg-lab/scRNA.seq.datasets/tree/master/R.

No additional normalization steps were performed, since the creators of the datasets choose a normalization method that was adequate for their data, and that transfer learning should work regardless of differences between normalization methods.

### XGBoost

XGBoost (“eXtreme Gradient Boosting”) is a supervised machine learning method that has received a great deal of attention in recent years [17]. In essence, XGBoost is an ensemble of regression trees, where the prediction for any given example is the sum of predictions from each tree in the ensemble. When learning the model, a regularized objective function is minimized, which is composed of two components, the prediction error and the complexity of the model. In XGBoost, emphasis is placed on approximating the splitting point for each split in the tree, with a dedicated logic for finding splits for missing data points, which makes the algorithm suitable for datasets with missing values, thereby obviating the need to populate missing values in the preprocessing stage.

The implementation specifics of XGBoost contain many performance optimizations, including built-in computation parallelism and cache-aware data access. All these properties make XGBoost a scalable tree boosting system that provides state-of-the-art results for a wide range of problems [17], including those of this study, where XGBoost proved to be an efficient and effective classification method. In this work we used the R implementation of XGBoost [31].
