# Correction: CaSTLe - Classification of single cells by transfer learning: Harnessing the power of publicly available single cell RNA sequencing experiments to annotate new experiments

**DOI:** 10.1371/journal.pone.0208349

**Published:** 2018-11-27

**Authors:** Yuval Lieberman, Lior Rokach, Tal Shay

There is an error in Fig 3. The authors have provided a corrected version here.

**Fig 3:**
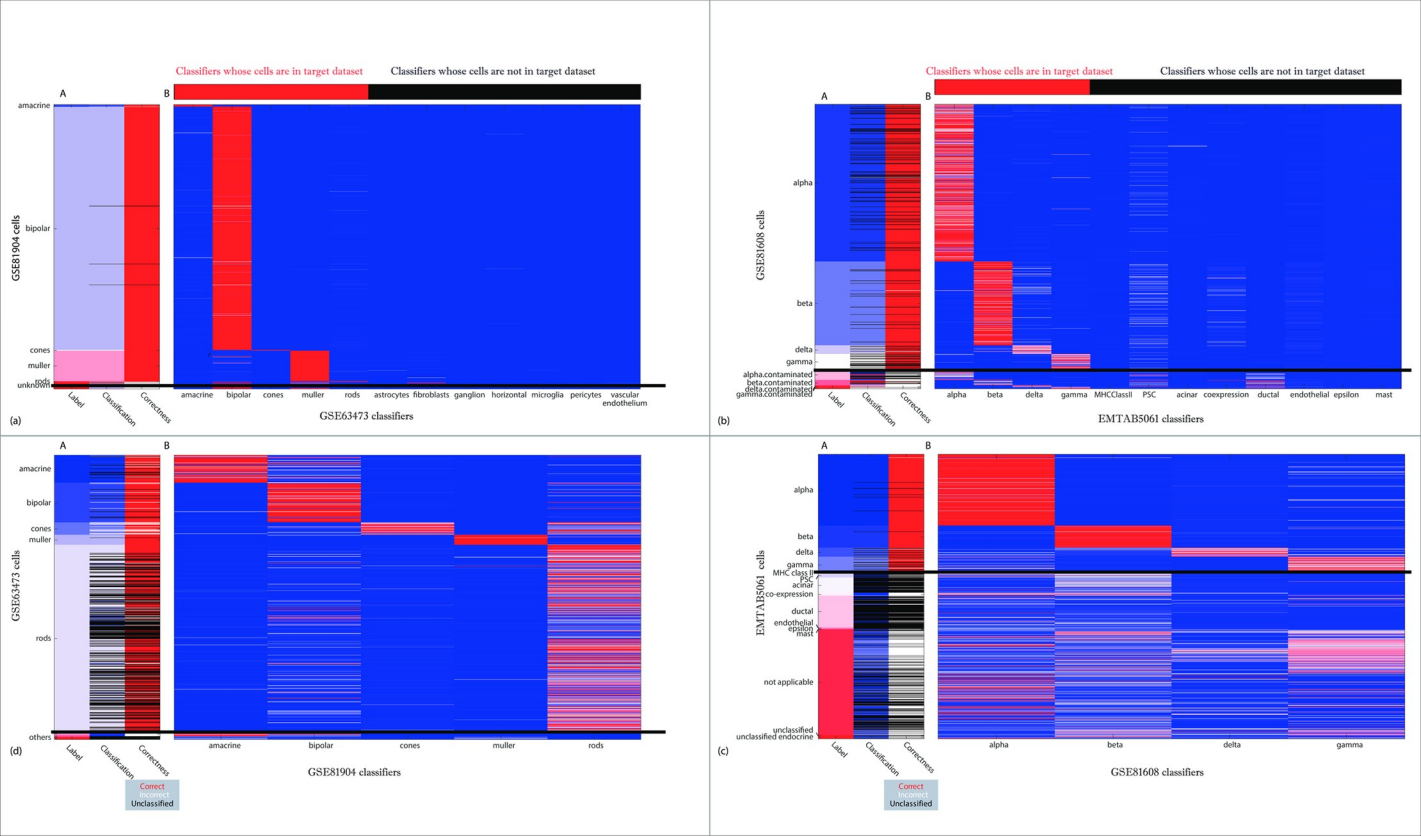
Classification of a target dataset by multiple binary classifiers. (A) The label of each cell in target dataset (left), the label each cell was given by the classifiers, black for unclassified (center), and the correctness of classification (right). (B) Heatmap of the scores given to each cell by each classifier, from zero (blue) to one (red). Horizontal black line separates the labels of those classifiers from labels for which classifiers were not trained. (a) Classification of target dataset GSE81904 by multiple binary classifiers built on source dataset GSE63473. Top bar shows which classifiers classified for labels that are in the target dataset. Note that many unknown cells were classified as bipolar or muller, which may be correct. (b) Classification of target dataset GSE81608 by multiple binary classifiers built on source dataset EMTAB5061. Top bar shows which classifiers classified for labels that are in the target dataset. Note that the labels that some of the seemingly incorrect results are very likely correct—the cells labeled as 'alpha contaminated' are classified as alpha or ductal, and same for beta, gamma and delta contaminated. (c) Classification of target dataset EMTAB5061 by multiple binary classifiers built on source dataset GSE81608. Note that many of the 'novel' cell types (acinar, ductal) were not classified, thus 'identified as novel'. Many of the incorrect cells are labeled 'coexpression' or 'not applicable' or 'unclassified', meaning that their classification may be correct. (d) Classification of target dataset GSE63473 by multiple binary classifiers built on source dataset GSE81904.
